# High-Acquisition-Rate Single-Shot Pump-Probe Measurements Using Time-Stretching Method

**DOI:** 10.1038/srep37614

**Published:** 2016-11-23

**Authors:** Masataka Kobayashi, Yasuo Minami, Courtney L. Johnson, Parker D. Salmans, Nicholas R. Ellsworth, Jun Takeda, Jeremy A. Johnson, Ikufumi Katayama

**Affiliations:** 1 Graduate School of Engineering, Yokohama National University, Yokohama, 240-8501 Japan; 2 Department of Chemistry and Biochemistry, Brigham Young University, Provo, Utah, 84602, USA

## Abstract

Recent advances of ultrafast spectroscopy allow the capture of an entire ultrafast signal waveform in a single probe shot, which greatly reduces the measurement time and opens the door for the spectroscopy of unrepeatable phenomena. However, most single-shot detection schemes rely on two-dimensional detectors, which limit the repetition rate of the measurement and can hinder real-time visualization and manipulation of signal waveforms. Here, we demonstrate a new method to circumvent these difficulties and to greatly simplify the detection setup by using a long, single-mode optical fiber and a fast photodiode. Initially, a probe pulse is linearly chirped (the optical frequency varies linearly across the pulse in time), and the temporal profile of an ultrafast signal is then encoded in the probe spectrum. The probe pulse and encoded temporal dynamics are further chirped to nanosecond time scales using the dispersion in the optical fiber, thus, slowing down the ultrafast signal to time scales easily recorded with fast detectors and high-bandwidth electronics. We apply this method to three distinct ultrafast experiments: investigating the power dependence of the Kerr signal in LiNbO_3_, observing an irreversible transmission change of a phase change material, and capturing terahertz waveforms.

Traditional ultrafast pump-probe measurements can require thousands to millions of laser shots to record a single time-dependent signal trace, which can make ultrafast measurements exceptionally time-consuming, and exclude the possibility of studying irreversible phenomena in many systems. Recent efforts have therefore been focused to allow the capture of an entire ultrafast signal waveform in a single probe shot, which is of particular advantage in terahertz (THz) and other varieties of ultrafast spectroscopy^1^,^2^,^3^. Single-shot techniques make possible measurements of photoinduced irreversible phenomena, asynchronous or chaotic phenomena, and phenomena with potential picosecond shot-to-shot jitter, such as the timing of pulses from a laser system synchronized to an x-ray free electron laser pulse^4^,^5^,^6^,^7^,^8^. Because of the increasing interest in and importance of understanding these and other phenomena, single-shot diagnostic tools are becoming more crucial in the field of ultrafast spectroscopy. Virtually all current single-shot ultrafast spectroscopic techniques, however, rely on two-dimensional detectors such as CCD and CMOS cameras, which limit the repetition rate of the measurement and hinder real-time visualization and manipulation of signal waveforms^9^,^10^,^11^,^12^.

In this work, we demonstrate a new method to circumvent these 2-D detector difficulties and to greatly simplify the single-shot setup by using a long, single-mode optical fiber and a fast photodiode (PD)^13^. This is a variation on spectrally-encoded single-shot measurements, where the probe pulse is linearly chirped, and the temporal signal is thus encoded in the probe spectrum^5^,^9^,^14^. Instead of observing the spectrum with a grating and a 2-D detector, the probe pulse and encoded temporal dynamics are further chirped to nanosecond time scales using the group velocity dispersion in the optical fiber, thus slowing down the ultrafast signal to time scales easily recorded with fast detectors and high-bandwidth electronics. Similar efforts have been made to accelerate the acquisition speed in ultrafast imaging measurements, such as the STEAM and STAMP techniques^15^, although in those cases the time-resolution and number of time points in the data are limited due to the huge number of data points required for imaging. Here, instead of imaging, we use all the probe wavelength components to better acquire high temporal resolution for time-resolved ultrafast spectroscopy, to greatly simplify the system and enable precise detection of sub-picosecond waveforms. Because the ultrafast analog waveform is temporally stretched to a nanosecond timescale, it becomes easy to realize high repetition rate measurements and can open ways to investigate the frontier of irreversible ultrafast phenomena.

The simple, yet powerful setups for three different experiments are shown in Fig. 1, in which the probe pulse is pre-chirped to map the time information to the wavelength. After passing the ultrafast probe pulse through a high dispersion SF11 glass rod in order to pre-chirp it, the probe pulse is then transmitted through a sample of interest, and subsequently directed to the input facet of a long fiber to further stretch each pulse into the nanosecond range. The nanosecond intensity profile from the optical fiber output was then read out by a fast PD and recorded using a high-bandwidth oscilloscope. Since our method only uses a single PD for the detection, it is now possible to obtain ultrafast waveforms at a high repetition-rate, essentially only limited by the laser repetition-rate and the acquisition rate of the oscilloscope^17^,^18^,^19^. We demonstrate in this paper the power of this novel single-shot detection scheme in three spectroscopic experiments: the observation and fast acquisition of pump-power dependent Kerr waveforms in ferroelectric LiNbO_3_, the measurement of the onset of a permanent photo-induced phase change in a chalcogenide alloy film, and the fast acquisition of THz waveforms via electro-optic sampling. Note that this probing scheme can be employed with great flexibility in virtually any ultrafast pump-probe experiment, and therefore allows us to measure a variety of ultrafast dynamics on a single-shot basis.

Lithium niobate (LiNbO_3_) is a ferroelectric crystal that, due to a number of favorable physical properties, is used in numerous photonic devices^20^,^21^. The loss of inversion symmetry allows and enhances the optical nonlinearities such as sum and difference frequency generation, the optical Kerr effect, and excitation of polaritons with ultrafast near-infrared (NIR) laser pulses^22^,^23^,^24^. These nonlinearities are important ingredients in designing future optoelectronic devices. In characterizing the nonlinear optical properties, evaluation of the power dependence is indispensable, which we demonstrate using the single-shot method proposed here. To demonstrate, we placed a crossed polarizer after the single-crystal LiNbO_3_, and recorded the polarization rotated signal induced by the pump pulse as shown in Fig. 1. The pump pulses are chopped at 500 Hz and each probe waveform (coincident with the pump) is normalized by the average of two probe profiles without the pump (one just before the pump and one just after) to minimize the fluctuation of the laser 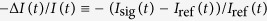. Figure 2a shows the measured pulse profiles at the detector with (*I*_sig_(*t*)) and without (*I*_sig_(*t*)) the pump pulse. The difference between these waveforms indicates the observation of a Kerr signal. As the repetition rate of the measurement was 500 Hz, we can obtain 500 data scans as shown in Fig. 2a within one second. A higher repetition rate laser system would lead to even faster data acquisition times; the maximum accumulation rate would then be determined by the trigger rate of the oscilloscope, which can exceed 100 kHz in modern high-bandwidth oscilloscopes.

The Kerr results are summarized in Fig. 2b (with a subset of traces in Fig. 2c), which displays recorded traces as the pump intensity is automatically ramped up during the measurement (the axis “Frames” corresponds each signal frame as the pump power is increased). The pump intensity is monitored with an additional PD recorded by the same oscilloscope. The power dependence of Kerr signal in LiNbO_3_ was evaluated from 2.3 mJ/cm^2^ to 31 mJ/cm^2^. As the pump fluence is increased, the Kerr response grows quadratically (see Fig. 2c,d), as is expected from the *χ*^(3)^ nonlinearity of the material in the homodyne configuration. The probe polarization after the sample becomes elliptical, making the signal proportional to the square of the pump intensity. With power dependent data obtained in one second, we estimate the magnitude of the nonlinear refractive index to be 4.2×10^−13^ esu, which agrees well with the previous reports on LiNbO_3_^25^. We note that the pulse duration of the Kerr signal was longer than that of the pump pulse, because of the signal distortions in the spectral encoding technique^14^, and the limited bandwidth (1 GHz) of the photodiode.

Perhaps the most useful advantage of this high-repetition rate single-shot measurement is the ability to study irreversible phenomena. To demonstrate this capability using the setup shown in Fig. 1, we examine the ultrafast laser-induced amorphization in the phase change material Ge_2_Sb_2_Te_5_ (GST), a famous material for optical storage devices^26^,^27^,^28^. The laser induced crystalline to amorphous phase change induces relatively high contrast in the optical constant, yet the ultrafast dynamics are less studied due to the difficulty in the measurement of the irreversible phase change^4^. Here the amplitude of transmitted probe light is monitored as the pump fluence is increased from 5.3 mJ/cm^2^ to 10.8 mJ/cm^2^, crossing the phase change threshold. Figure 3a shows the observed probe profiles as a function of time (the left panel), and transmission change calculated by the pulses with and without the pump pulse (−Δ*I(t*)/*I(t*) – the right panel). Again, the vertical axis in this figure corresponds to the time of the measurement (as the pump fluence is increased), and each frame is captured at 500 Hz acquisition rate.

We see a clear change of the probe intensity and the ultrafast profile at 190 frame, which correspond to the threshold (8.1 mJ/cm^2^) where the phase change takes place^4^. After this threshold, the transmitted probe intensity increases gradually, indicating increased amount of the amorphous phase in the probed area. With this phase change, we also observe the change in the ultrafast waveform, which is highlighted in the right as well as in the upper panel of Fig. 3b. The change in dynamics is consistent with the previous reports on the ultrafast dynamics of crystalline and amorphous phases as shown by the dashed lines in these figures^4^,^29^. Even though there are oscillatory signals seen in this figure that are caused by the limited time resolution of the spectral encoding technique^14^, there remains fairly good agreement with the previous reports^4^,^29^.

In Fig. 3b, comparison of the probe intensity and the pump intensity is plotted for each frame. The probe intensity starts increasing after the pump intensity reaches the phase change threshold. Interestingly, even though the pump intensity reaches its maximum at frame 290, the probe intensity continues to increase for some time, indicating an accumulative effect. The reason for this accumulative effect may be related to the ultrafast dynamics change above and below the threshold, which are featured in the right of Fig. 3. The ultrafast profiles above the threshold could be reproduced by the superposition of these ultrafast profiles in crystalline and amorphous phases, whose ratio will be seen in the transient transmittance at 1 ps as shown in Fig. 3b. The evolving dynamics indicate a modification of the excited material relaxation pathway above the threshold that makes the relaxation to the photoinduced amorphous phase dominant. The modification may result from the change of the potential landscape to accelerate the relaxation to the photoinduced phase, inducing this accumulative effect^30^. Note that this measurement cannot be repeated by traditional means because the phase change is irreversible. The phase change to the amorphous state may only be reversed if an erase pulse is shined on the sample, or the temperature is cycled, thus clearly demonstrating the strength of this fiber based method for the dynamical studies.

Finally, we apply this technique to the terahertz spectroscopy and examine the reliability of measurements of more complicated waveforms. Broadband THz radiation is generated via optical rectification of 1200 nm light in the organic THz generation crystal OH1^31^, in which the water vapor absorption and the OH1 phonons imprint modulations in the generated terahertz waveforms. The generated THz radiation is detected using an electro-optic sampling method as shown in Fig. 1. Figure 4a illustrates the observed output pulse-profiles from the photodiode with and without terahertz pulses, for different relative delay times between the terahertz pulse and probe pulse. We clearly see the difference of the waveforms induced by the terahertz pulses. By normalizing the terahertz signal by a reference probe pulse (grey lines in Fig. 4a), Δ*I(t*)/*I(t*), the THz waveform can readily be compared to that recorded with a conventional stage scan method (the dark line in Fig. 4b, recorded by stepping a delay stage to change the relative delay between THz and a ~100 fs 800 nm probe pulse).

In order to test the signal reconstruction and to calibrate the time-scaling factor, we stepped the relative delay, increasing the relative path-length by a known amount, between the pre-stretched probe and THz pulses and recorded scans at 5 different positions (labeled SS 1–5 in Fig. 4). The good agreement of these waveforms shows the effectiveness of the simple calibration method and demonstrates the promising capability of this fiber single-shot technique to record complex THz waveforms, potentially at exceedingly high acquisition rates. Additionally, the ability to observe the complete THz waveform in real time (see particularly the trace labeled SS3 in Fig. 4a), allows for easy optimization of experimental alignment.

In summary, we demonstrated novel single-shot high-repetition-rate measurements of ultrafast and terahertz waveforms by only using a long optical fiber, a single photodiode, and an oscilloscope. We realized the fast acquisition of Kerr waveforms in LiNbO_3_, an irreversible ultrafast phase change in a GST thin film, and terahertz waveforms that agree well with those obtained by using a conventional stage scan method. Future work will aim to increase the temporal window of the single-shot detection, as well as to improve the time resolution, which will further enhance the variety of applications using this fast, simple, single-shot measurement scheme, for example, in fundamental sciences of irreversible and dynamic phenomena, terahertz imaging, information and communication technologies, material characterization, and so on.

## Methods

In the Kerr and GST experiments, the output of a Ti:sapphire amplifier with 40-fs pulse duration, 1-kHz repetition rate, 1.4-mJ output power and 800-nm center wavelength is used. The laser was divided into pump and probe beams. The pump was chopped at 500 Hz, and the intensity was rapidly scanned by computer-controlled rotation of a half-waveplate placed before a polarizer. The probe was passed through a 150-mm-long SF11 glass rod twice to induce positive chirp and ~4-ps pulse duration. The pump and probe pulses were then focused on a sample with polarizations oriented 45 degrees with respect to each other. Next the polarization rotated signal or the transmitted probe pulses were input into a 3-km-long single-mode fiber (Nufern, 780-OCT), and the intensity profile was recorded using a 1-GHz photodiode (ThorLabs DET02AFC) and a 12.5-GHz real-time oscilloscope (Tektronics DPO71254C). The signal intensity was kept low to not cause any nonlinearity in the fiber that may distort the signal waveform. The samples were a 1-mm-thick x-cut LiNbO_3_ single crystal, or a Ge_2_Sb_2_Te_5_ thin film with the thickness of 20 nm deposited on a sapphire substrate.

The terahertz experiments were similar in construction, albeit utilizing a different wavelength. A 1200 nm ~100 fs laser pulse derived from an optical parametric amplifier was split into pump and probe pulses. The pump pulses were used to generate broadband THz pulses via optical rectification in the organic crystal OH1. The probe pulses were pre-stretched to approximately 2 ps by passing through a 150 mm SF11 glass rod, and then focused collinearly with the THz pulses to a 300 μm thick (110) GaP electro-optic crystal. Next, after passing through a quarter-waveplate oriented at 45 degrees to the initial probe polarization, and a polarizer, the probe beam was focused and input into a 3-km single-mode optical fiber from Mitsubishi (Optiflex FSFC2/9S-S-V/1–3 K). The resulting nanosecond probe profiles were recorded using an Agilent Infiniium 86100 A oscilloscope with 20 GHz bandwidth optical input module. The pump/probe wavelength was selected to ensure significant stretching in the single mode optical fiber resulting in a probe intensity profile of a few ns duration. The relative delay between pump and probe was changed, and the data compared to electro-optic traces recorded using a conventional stage scan technique with ~100 fs 800 nm probe pulses.

## Additional Information

**How to cite this article**: Kobayashi, M. *et al*. High-Acquisition-Rate Single-Shot Pump-Probe Measurements Using Time-Stretching Method. *Sci. Rep.* **6**, 37614; doi: 10.1038/srep37614 (2016).

**Publisher’s note:** Springer Nature remains neutral with regard to jurisdictional claims in published maps and institutional affiliations.

## Figures and Tables

**Figure 1:**
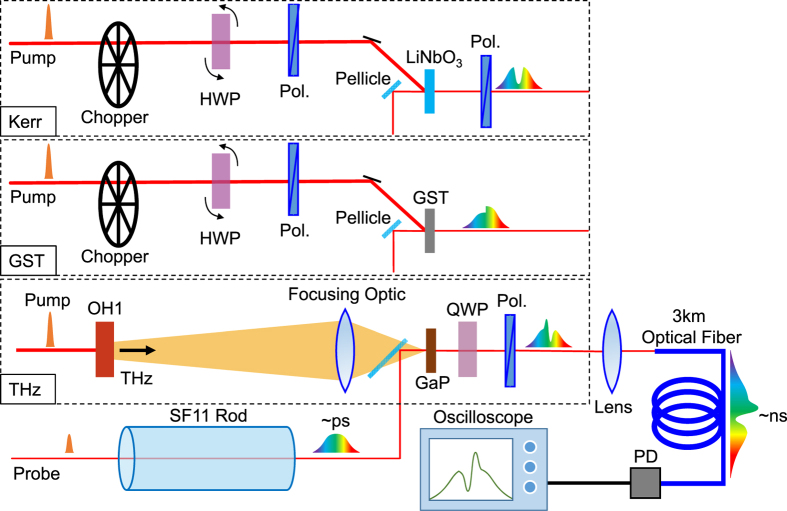
Experimental setups for the single-shot ultrafast measurements. For the Kerr measurement, we placed crossed polarizers (Pol.) before and after the sample to detect the polarization rotated signal. We rotated the half waveplate (HWP) during the measurement, and the intensity of the probe laser was then tuned from 2.3 mJ/cm^2^ to 31 mJ/cm^2^. For the GST measurement, we monitored the transmission change induced by the pump pulse, whose intensity was tuned with the similar setup for Kerr measurement. For the THz demonstration, we generated terahertz waves with an OH1 organic nonlinear crystal, and the electric field was detected using a conventional EO sampling method with a GaP crystal, a quarter waveplate (QWP) and a polarizer. In all the measurements, probe pulses were pre-chirped using SF11 glass rod. The output was sent to a 3-km optical fiber that was connected to a fast photodiode. The signal traces were recorded using a real-time oscilloscope with sufficient bandwidth.

**Figure 2:**
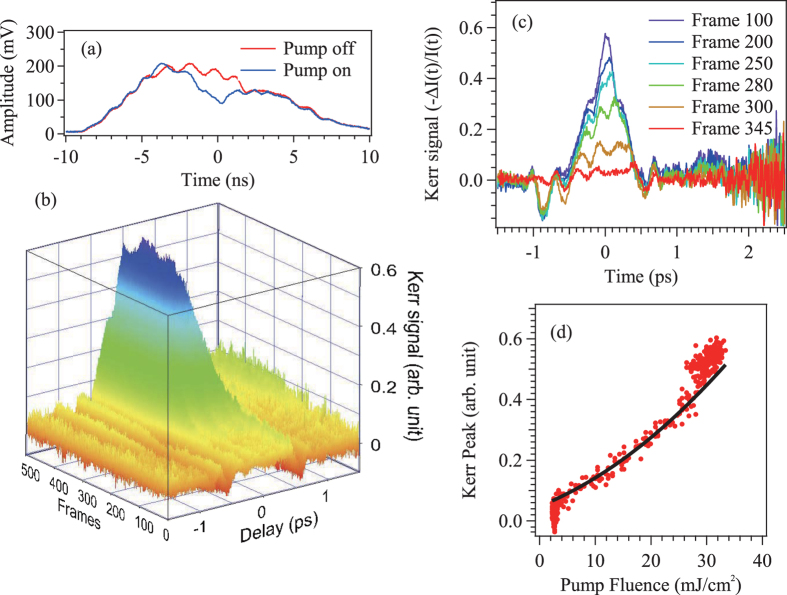
An intensity dependence of a Kerr measurement in LiNbO_3_. (**a**) Slowed-down signal traces with and without a pump pulse observed directly at the photodiode. Small modulation of the spectrum may be due to an unintentional echo pulses generated in some optics in the setup, which does not severely affect the signal profiles. (**b**) 500 signal traces (−*ΔI/I*) captured while changing the pump intensity. The time axis is calibrated by measuring two waveforms with changing the pump delay time. Every waveform is normalized by the average of two probe profiles without the pump as described in the text. (**c**) Slices of the Kerr waveforms from (**b**). (**d**) The power dependence of the peak intensity at the time origin extracted from the data shown in (**b**). The black solid line indicates the quadratic fitting expected for the Kerr nonlinearity.

**Figure 3:**
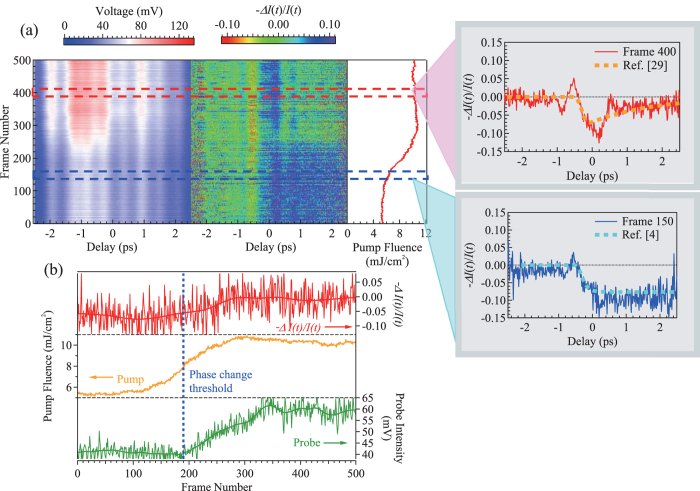
Fast acquisition of pump and single-shot probe data on the phase change material GST. (**a**) Intensity profiles of the probe pulses (left) and the normalized transmission change (−*ΔI/I*) (middle). The right-most trace indicates the pump intensity simultaneously monitored using the same oscilloscope. The slices of the ultrafast waveforms are shown in red (frame 400 in the amorphous phase) and blue (frame 150 in the crystalline phase) at the right side of the figure. The blue and orange dashed lines show the crystalline-to-amorphous phase change and the relaxation dynamics in the amorphous phase, respectively, which are taken from the data in refs 4 and 29. (**b**) The transmission change at 1 ps (top), pump (middle), and probe (bottom) intensities as a function of the frame number. Solid lines in the figures indicate the average of 10 frames around. Since the measurement was made at 500 Hz, the horizontal axis corresponds to the time of the measurement.

**Figure 4:**
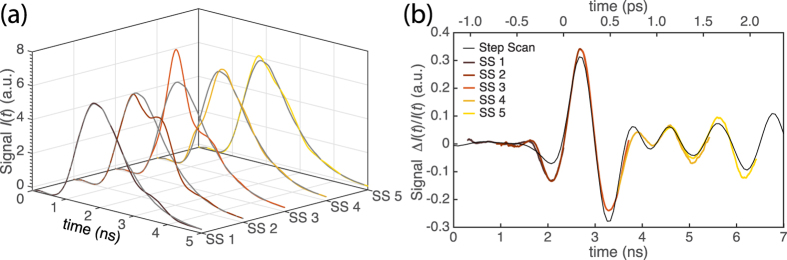
The results of the single-shot terahertz measurements. (**a**) The probe profiles with and without the terahertz waves as the relative delay between THz and probe pulses was changed. (**b**) THz time traces compared to the step scan method. The black line indicates the time traces captured using the traditional step-scan method. The data with five different relative delay times are shown to demonstrate the precise measurement of the terahertz waveforms.
